# Sexual dimorphism of monocyte transcriptome in individuals with chronic low-grade inflammation

**DOI:** 10.1186/s13293-021-00387-y

**Published:** 2021-07-28

**Authors:** Jisun So, Albert K. Tai, Alice H. Lichtenstein, Dayong Wu, Stefania Lamon-Fava

**Affiliations:** 1 Cardiovascular Nutrition Laboratory, Jean Mayer USDA Human Nutrition Research Center on Aging at Tufts University, Boston, MA USA; 2 Department of Immunology, Tufts University School of Medicine, Boston, MA USA; 3 Nutritional Immunology Laboratory, Jean Mayer USDA Human Nutrition Research Center on Aging at Tufts University, Boston, MA USA

## Abstract

Sexual dimorphism in the immune system is evidenced by a higher prevalence of autoimmune diseases in women and higher susceptibility to infectious diseases in men. However, the molecular basis of these sex-based differences is not fully understood. We have characterized the transcriptome profiles of peripheral blood monocytes from males and postmenopausal females with chronic low-grade inflammation. We identified 41 sexually differentially expressed genes [adjusted *p* value (FDR) < 0.1], including genes involved in immune cell activation (e.g., *CEACAM1*, *FCGR2B*, and *SLAMF7*) and antigen presentation (e.g., *AIM2*, *CD1E*, and *UBA1*) with a higher expression in females than males. Moreover, signaling pathways of immune or inflammatory responses, including interferon (IFN) signaling [z-score = 2.45, -log(*p*) = 3.88], were found to be more upregulated in female versus male monocytes, based on a set of genes exhibiting sex-biased expression (*p* < 0.03). The contribution of IFN signaling to the sexual transcriptional differences was further confirmed by direct comparisons of the monocyte sex-biased genes with IFN signature genes (ISGs) that were previously curated in mouse macrophages. ISGs showed a greater overlap with female-biased genes than male-biased genes and a higher overall expression in female than male monocytes, particularly for the genes of antiviral and inflammatory responses to IFN. Given the role of IFN in immune defense and autoimmunity, our results suggest that sexual dimorphism in immune functions may be associated with more priming of innate immune pathways in female than male monocytes. These findings highlight the role of sex on the human immune transcriptome.

**Supplementary Information:**

The online version contains supplementary material available at 10.1186/s13293-021-00387-y.

## Introduction

Sex dimorphism is one of the critical factors contributing to immunological variability among individuals and has been under-appreciated in the majority of immunology studies [1, 2]. Phenotypic differences in immune-related diseases between the sexes provide evidence of this dimorphism: while men have a higher susceptibility to a variety of pathogens leading to an increased frequency of infectious diseases [3], women have a higher rate of autoimmune diseases [4]. Females, compared to males, show a stronger humoral and cell-mediated immunity [5, 6], as demonstrated by higher levels of immunoglobulins [7] as well as stronger antibody responses to viral vaccines [8]. Systemic lupus erythematosus (SLE), the autoimmune disease with the most striking female-biased prevalence [9], is characterized by overproduction of autoantibodies, resulting in inflammation and organ damage [10].

There are several hypotheses explaining the sex differences in immune function. Differences in sex steroid hormone concentrations [11] and sex chromosomes [12] play a prevailing role in genomic regulation of the immune system. Upon receptor binding, sex steroid hormones exert biological effects on immune cells by influencing signaling pathways such as nuclear factor kappa B (NF-κB), c-Jun, and interferon regulatory factors (IRF) in various lymphoid tissues as well as circulating immune cells [13]. Genes unique to the Y chromosome or additional copies of X chromosome genes that escape X-inactivation result in differential gene expression between males and females [12]. For example, some immune-related genes located on the X chromosome such as *TLR7* and *CD40LG* are expressed at higher levels in females than males [14, 15], possibly as a result of imperfect X-silencing. Particularly, duplication of *TLR7* has been suggested as an underlying mechanism for the higher susceptibility to SLE in mice [14]. Also, sex-specific genetic polymorphisms on autosomal genes and epigenetic controls may contribute to sexual differential gene expression [2].

Transcriptome analyses have provided a foundation for a better understanding of the molecular basis of the phenotypic immunological differences between the sexes [16–19]. Sexually differential gene expression has been documented in whole blood and peripheral blood mononuclear cells (PBMC) [20–23]. However, due to limitations in the ability to control for different proportions of immune cell subsets between the sexes [21–23], there is an incomplete understanding of the transcriptional sex dimorphism manifested by different bloodborne cell types. Data from mouse microglial cells indicate only a limited number of sex chromosome genes differentially expressed between the sexes [24]. In contrast, mouse bone marrow-derived macrophages (BMDM) displayed a moderate sex-dependent effect in a large number of genes [25]. Using samples from the Immunological Genome Project (ImmGen, http://www.immgen.org), immune transcriptome profiling of 11 different murine immune cell types at baseline and after immune challenge demonstrated that only macrophages exhibited sex dimorphism [19]. In particular, unstimulated macrophages showed a female-biased expression of interferon (IFN)-responsive genes that was further increased upon IFN stimulation, accompanied by stronger antiviral and inflammatory responses [19]. The greater activation of IFN pathways upon stimulation in these cells suggests that females may be equipped with a more vigilant immune defense system that exerts a more vigorous immune response against external or internal challenges. However, considering the strong correlation between IFN overexpression and autoimmune diseases such as SLE [26], elevated expression of genes in these pathways in females in the absence of a clear threat signal is suggested to be one of the major factors contributing to a higher prevalence of autoimmunity in females compared to males.

In light of the transcriptional sex dimorphism observed in mice, it is of interest to explore cell-type-specific sex effects on basal immune transcriptomes in humans. While conducting a study on the effects of omega-3 fatty acid supplementation on inflammation in individuals with chronic low-grade inflammation [27], we observed evidence of sex dimorphism in monocyte transcriptome. Therefore, the aim of this study, as a secondary analysis of the parent study, was to identify differentially expressed genes on the basis of sex in human monocytes obtained from peripheral blood at baseline and to characterize the biological pathways, particularly immune-related pathways, associated with the transcriptional sex dimorphism. We hypothesized different basal monocyte transcriptome profiles between males and females with overexpression of immune-related genes, specifically IFN responsive genes, in females.

## Materials and methods

### Study participants

This study is part of a randomized, double-blind, crossover clinical trial (registered at clinicaltrials.gov as NCT02670382) assessing the effect of omega-3 fatty acid supplementation on inflammation in individuals with chronic low-grade inflammation [27]. Data for the current study were obtained at the end of the lead-in control phase, defined as baseline. Participants were recruited through advertising in local newspapers. Males and postmenopausal females aged between 50 and 75 years were screened by a priori defined inclusion criteria for chronic low-grade inflammation including serum high-sensitivity C-reactive protein (hs-CRP) concentration ≥ 2 μg/mL, fasting plasma triglyceride concentration between 90 and 500 mg/dL, and having at least one of the following characteristics: abdominal obesity (waist circumference ≥ 102 cm in men and ≥ 89 cm in women), hypertension (blood pressure ≥ 130/80 mmHg or use of anti-hypertensive medications), or fasting plasma glucose ≥ 100 mg/dL, but otherwise healthy. Twenty-one participants completed the study, and 19 (9 males and 10 females) who had monocyte samples with high purity were included in the current study for determining the transcriptional sexual dimorphism in peripheral blood monocytes. The racial distribution was 14 Whites, 3 Blacks, 1 Hispanic, and 1unknown.

### Blood collection, monocyte isolation, and RNA extraction

Venous blood was drawn after a 12-h overnight fast in sodium citrate Vacutainer Cell Preparation Tubes (Beckton Dickinson, Franklin Lakes, NJ) for PBMC isolation. All subtypes of monocytes including classical (CD14^++^/CD16^−^), non-classical (CD16^++^/CD14^+^), and intermediate (CD16^+^/CD14^++^) were further isolated from the PBMC fraction by a negative selection method using antibody-coupled magnetic beads (Miltenyi Biotec, Bergisch Gladbach, Germany). The flow-through cells were centrifuged at 300×*g* for 10 min at 22 °C and stored at − 80 °C until further analyses. Total RNA was isolated using QIAshredder and RNeasy Mini kit (both from QIAGEN, Hilden, Germany) according to the manufacturer’s instructions. Isolated RNA was treated on-column with RNase-free DNase (QIAGEN, Hilden, Germany) to eliminate genomic DNA contamination. RNA quality was assessed using a Fragment Analyzer (Agilent, Santa Clara, CA).

### RNA sequencing data generation, processing, and analysis

RNA samples that passed quality checks were used as input to prepare RNA-Seq library using Illumina TruSeq stranded mRNA kit (Illumina, San Diego, CA) per manufacturer instruction. Briefly, mRNA was enriched via polyA selection from input total RNA. Enriched mRNA was then fragmented, followed by first cDNA synthesis with random priming, and second-strand cDNA synthesis with dUTP. The 3′ adenylates were added to the double-stranded cDNA, followed by adaptor ligation and second strand removal and amplification. The molar concentration and size distribution of resultant libraries were assessed on a Fragment Analyzer (Agilent, Santa Clara, CA). Libraries were sequenced on an Illumina HiSeq 2500 sequencer (Illumina, San Diego, CA) with High Output V4 chemist and 50 base-pair single-end reads format. Raw data in FASTQ format were processed for quality control using FastQC (https://www.bioinformatics.babraham.ac.uk/projects/fastqc/) and then mapped to the human genome (USC hg38) using HISAT v2.1 (http://www.ccb.jhu.edu/software/hisat/index.shtml). The mapped reads to genes were quantified by featureCounts (http://subread.sourceforge.net/), and raw count data were normalized by the median of ratios method using DESeq2 package from Bioconductor (https://bioconductor.org/packages/release/bioc/html/DESeq2.html). For principal component analysis (PCA) and heatmap presentation, the normalized counts were variance stabilized using a regularized log transformation.

Monocyte purity was assessed by cell-type-specific gene expression, which led to the exclusion of two female participants who displayed abnormally high counts of *CD3* and *CD8* (markers for T cells), *CD19* (a marker for B cells), and *NCAM1* (a marker for natural killer, or NK cells). The count data were re-normalized based on the remaining 19 samples.

Differential gene expression between the sexes was compared using Wald tests of the DEseq2 package followed by the Benjamini-Hochberg false discovery rate (FDR) correction for multiple comparisons [28]. The difference in fold change was calculated as a ratio of expression values in females versus males and then log_2_ transformed. Genes with FDR of 0.1 or less were termed as differentially expressed genes. The analysis was performed using R 3.6.2.

### Pathway analysis

To determine canonical signaling pathways that contained the differentially expressed genes, we performed a pathway analysis using ingenuity pathway analysis (IPA; v 9.0, Qiagen, Redwood City, CA). The z-score, which is to infer the activation state of implicated biological pathway/function, was determined by the observed gene regulation (“up” or down”) and a literature-derived direction of effect of the gene to the pathway (“activating” or “inhibiting”). Pathways with absolute |z-score| ≥ 1 and *p* < 0.05 (calculated by a right-tailed Fisher’s exact test) were considered significant.

### Interferon signature genes

Mostafavi et al. [29] charted the transcriptional responses induced by IFNα in mouse macrophages from the ImmGen and identified macrophage-specific interferon signature genes ISGs (MF-ISGs) that were upregulated with > 2-fold increase and FDR < 0.1 [19]. To compare human monocyte sex-biased genes identified in the present study with the mouse MF-ISGs, all mouse gene symbols of the MF-ISGs were translated to their human orthologs using ENSEMBL BioMart data mining tool (https://useast.ensembl.org/info/data/biomart/index.html) [30]. The fold change distribution of the human MF-ISG orthologs was compared to that of all other mapped genes by two-sided Welch’s *t* test.

We also compared our monocyte sex-biased genes to the expanded list of 628 ISGs constituting an IFN transcriptional regulatory network that was computationally built based on 1,398 human and mouse datasets by Mostafavi et al. [29]. The direction of fold changes of the ISGs from our monocytes was tested by a one-sample two-sided *t* test with the Bonferroni correction [31].

## Results

### Participant characteristics

Consistent with the eligibility criteria, study participants had chronic low-grade inflammation as indicated by a median hs-CRP concentration greater than 3 μg/mL (Table 1). Participant characteristics and measures of inflammation were similar between females and males, with the exception of diastolic blood pressure which was lower in females than in males (two-sided *t* test *p* < 0.02). Similarly, monocytes, as a percent of total leukocytes in peripheral blood, were similar between the sexes.

**Table 1:** Characteristics of study participants

Variables	Males (*n* = 9)	Females (*n* = 10)	*p*
Age (years)	59 ± 6	63 ± 6	0.20
Body mass index (kg/m^2^)	31.6 ± 5.3	33.6 ± 7.5	0.52
Waist circumference (cm)	111 ± 13	101 ± 17	0.17
Systolic blood pressure (mmHg)	129 ± 7	127 ± 24	0.76
Diastolic blood pressure (mmHg)	85 ± 6	73 ± 12	0.02
Total leukocyte count (1000/uL)	6.5 ± 1.3	5.7 ± 1.7	0.28
Lymphocyte proportion (% of leukocytes)	28 ± 5	31 ± 8	0.24
Monocyte proportion (% of leukocytes)	7.9 ± 1.8	7.9 ± 1.9	0.99
Serum inflammatory markers^†^			
Hs-CRP (μg/mL)	3.39 ± 2.43	3.18 ± 1.93	0.55
TNF-α (pg/mL)	2.04 ± 0.68	2.54 ± 0.75	0.12
IL-6 (pg/mL)	0.83 ± 0.68	0.75 ± 0.49	0.72
MCP-1 (pg/mL)	288 ± 136	302 ± 74	0.59

Values are means ± SDs (or ^†^medians ± interquartile ranges)

*P* values were determined by two-sample student *t* test or Wilcoxon test

*Hs*-*CRP* high-sensitivity C-reactive protein

### Monocyte-specific sex signature genes

**Fig. 1:**
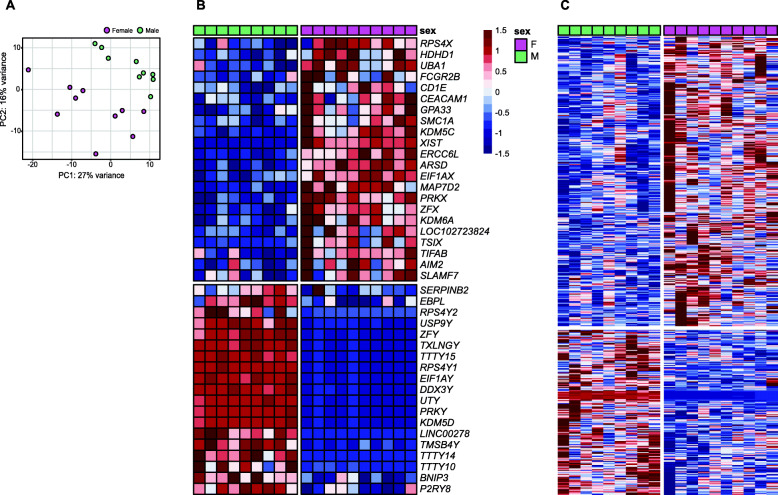
Monocyte transcriptional sex dimorphism. **a** Principal component analysis (PCA) of peripheral blood monocytes of males and females (green and pink) based on 26,485 genes. **b**, **c** Heatmaps of relative expression of 41 sexually differentially expressed genes (FDR < 0.1) and 565 sex-biased genes (*p* < 0.03) in peripheral blood monocytes from male (green) and female (pink) participants. A horizontal gap separates female- from male-biased genes, and a vertical gap separates male and female participants

Monocyte transcriptional sex dimorphism was clearly observed, as shown in the separation of the samples based on sex in the PCA plot (Fig. 1A). Of the 41 sexually differentially expressed genes identified, the expression of approximately half (22 genes, 54%) was higher in females than males. Of the 22 genes, 7 were autosomal and 15 genes were X-linked. These genes are involved in immune cell activation (*CEACAM1*, *FCGR2B*, and *SLAMF7*) and antigen-recognition or presentation (*AIM2*, *CD1E*, and *UBA1*), suggesting enhanced activation of innate and adaptive immune responses in monocytes from females compared to males. Expression of 19 genes (46%) was higher in males than females. These included three autosomal genes (*SERPINB2*, *BNIP3*, and *EBPL*) and 16 Y-linked genes. The overall median absolute log_2_ fold difference in females versus males was 1.38 (Fig. 1B, Supplementary Table S1).

### Increased activation of immune responses in monocytes

To gain further insight into the biological pathways that are associated with the transcriptional sexual dimorphism in monocytes, we selected genes that were significant at *p* < 0.03 for the sexual difference (360 female-biased and 205 male-biased genes; Fig. 1C, Supplementary Table S2) and mapped them to the canonical pathways of the IPA database. Out of the 11 canonical pathways that were significantly upregulated in females (z-score ≥ 1, -log(*p*) > 1.30 by Fisher’s exact test), most pathways (8/11, 73%) were involved in immune or inflammatory responses (Table 2). IFN signaling was the top (z-score = 2.45, -log(*p*) = 3.88) among the 11 female-biased pathways, consistent with its documented association with female-biased autoimmunity [23, 32]. Genes involved in IFN signaling (IFN-induced protein with tetratricopeptide repeats [*IFIT*] 1-3, IFN-induced transmembrane protein 1 [*IFITM1*], Janus kinase 2 [*JAK2*], signal transducer and activator of transcription 1 [*STAT1*]), antigen-presentation (CD1 molecules [*CD1B, CD1C*], major histocompatibility complex, class II, DO alpha [*HLA-DOA*], major histocompatibility complex class II, DR beta 5 [*HLA-DRB5*]), and immune cell activation (C-C motif chemokine receptor 5 [*CCR5*], CD274 molecule [*CD274*], CD40 molecule [*CD40*], Fc fragment of IgG receptor IIb [*FCGR2B*]) were principally implicated in the other significant pathways (i.e., T helper type 1 (Th1) pathway, dendritic cell maturation, crosstalk between dendritic cells and NK cells, and SLE in B cell signaling pathway) as well. In addition to the 11 significant female-biased pathways, we identified two male-biased pathways with borderline significance: pathways related to programmed cell death protein 1 (PD-1), programmed cell death-ligand1 (PD-L1) cancer immunotherapy (z-score = − 0.82, -log(*p*) = 2.01) and p53 signaling (z-score = − 0.45, -log(*p*) = 1.64).

**Table 2:** Top IPA biological pathways (|z-score| ≥ 1, *p* < 0.05) of sex-biased genes (*n* = 565, *p* < 0.03) in peripheral blood monocytes

Top pathways	Activation	Molecules	Functions and diseases	*p*
Interferon Signaling	Up	*IFIT1*, *IFIT3*, *IFITM1*, *JAK2*, *MX1*, *STAT1*	Cellular Immune Response; Cytokine Signaling	1.33E-04
PI3K Signaling in B Lymphocytes	Up	*CD180*, *CD40*, *CD79A*, *DAPP1*, *FCGR2B*, *IRS2*, *ITPR2*, *PIK3CG*, *PLCH1*, *PLCH2*	Cellular Immune Response	1.10E-03
Sperm Motility	Up	*ABL2*, *AXL*, *EPHB3*, *FLT1*, *GUCY1A1*, *ITPR2*, *JAK2*, *MAP2K6*, *MAP3K11*, *PLAAT4*, *PLAAT5*, *PLCH1*, *PLCH2*	Organismal Growth and Development	1.60E-03
Th1 Pathway	Up	*CCR5*, *CD274*, *CD40*, *HLA-DOA*, *HLA-DRB5*, *JAK2*, *MAP2K6*, *PIK3CG*, *STAT1*	Cellular Growth and Proliferation and Development; Cellular Immune Response; Cytokine Signaling; Pathogen-Influenced Signaling	1.60E-03
Dendritic Cell Maturation	Up	*CD1B*, *CD1C*, *CD40*, *FCGR2B*, *HLA-DOA*, *HLA-DRB5*, *JAK2*, *PIK3CG*, *PLCH1*, *PLCH2*, *STAT1*	Cellular Immune Response; Cytokine Signaling; Pathogen-Influenced Signaling	2.83E-03
Type I Diabetes Mellitus Signaling	Up	*FAS*, *HLA-DOA*, *HLA-DRB5*, *ICA1*, *JAK2*, *MAP2K6*, *STAT1*	Apoptosis; Disease-Specific Pathways	1.24E-02
Crosstalk between Dendritic Cell and Natural Killer Cells	Up	*CD40*, *FAS*, *HLA-DRB5*, *KIR3DL2*, *TLN2*, *TNFSF10*	Cellular Immune Response	1.48E-02
UVA-Induced MAPK Signaling	Up	*PARP12*, *PARP9*, *PIK3CG*, *PLCH1*, *PLCH2*, *STAT1*	Cellular Stress and Injury	2.27E-02
Adrenomedullin Signaling Pathway	Up	*ADCY5*, *GUCY1A1*, *ITPR2*, *MAP2K6*, *PIK3CG*, *PLCH1*, *PLCH2*, *RAMP3*, *SOX15*	Cardiovascular Signaling; Cellular Growth, Proliferation and Development; Cellular Stress and Injury	3.35E-02
Pancreatic Adenocarcinoma Signaling	Up	*CDK2*, *E2F2*, *E2F6*, *JAK2*, *PIK3CG*, *STAT1*	Cancer; Disease-Specific Pathways	3.57E-02
Systemic Lupus Erythematosus in B Cell Signaling Pathway	Up	*CD40*, *CD79A*, *FCGR2B*, *IFIT2*, *IFIT3*, *JAK2*, *LILRA6*, *PIK3CG*, *PLAAT4*, *STAT1*, *TNFSF10*	Cellular Immune Response; Disease-Specific Pathways	4.59E-02
PD-1, PD-L1 Cancer Immunotherapy Pathway^2^	Down (*z* = − 0.82)	*CD274*, *CDK2*, *HLA-DOA*, *HLA-DRB5*, *JAK2*, *PDCD1LG2*, *PIK3CG*	Cancer; Cellular-Immune Response	9.74E-03
p53 signaling^†^	Down (*z* = − 0.45)	*CDK2*, *FAS*, *PIK3CG*, *PML*, *THBS1*, *TP53I3*	Cancer; Ingenuity Toxicity List Pathways	2.27E-02

The direction of activation is based on the z-score calculated as females relative to males

^†^The most affected among the pathways downregulated (− 1 < z-score < 0, *p* < 0.05) in females relative to males

### Comparison of IFN signature and regulatory pathways

**Fig. 2:**
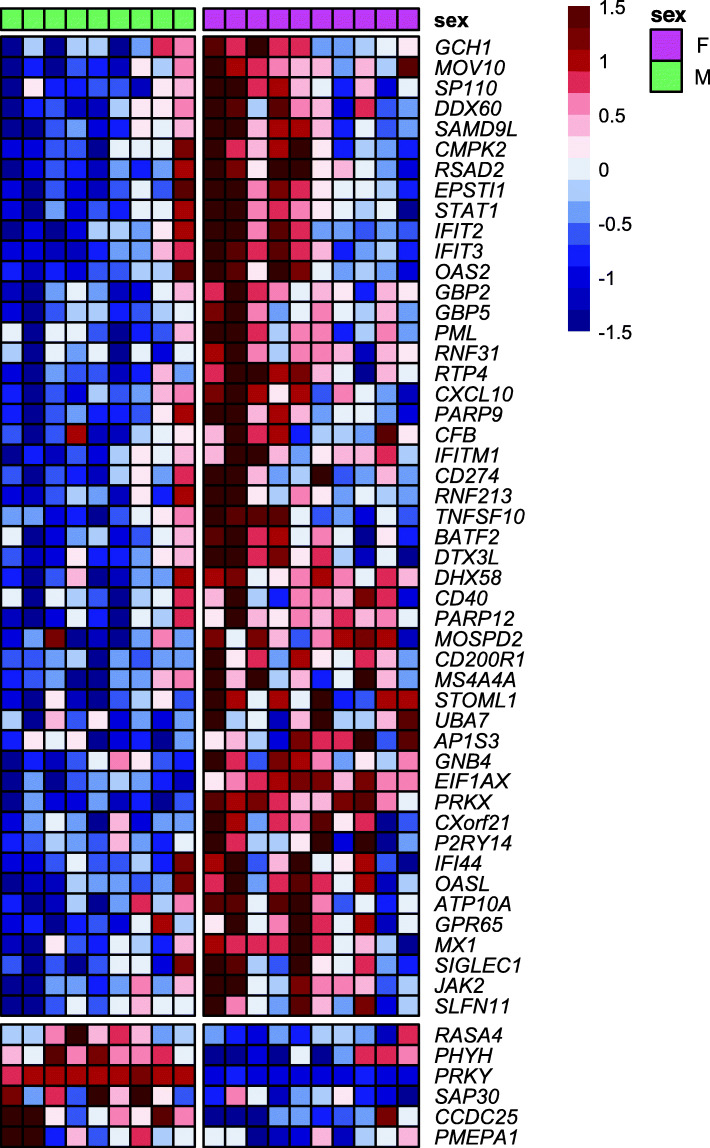
Heatmap of relative expression of 54 macrophage-specific IFN signature genes (MF-ISGs) that are also identified as sex-biased genes in peripheral blood monocytes. A horizontal gap separates female- from male-biased genes, and a vertical gap separates male and female participants

**Fig. 3:**
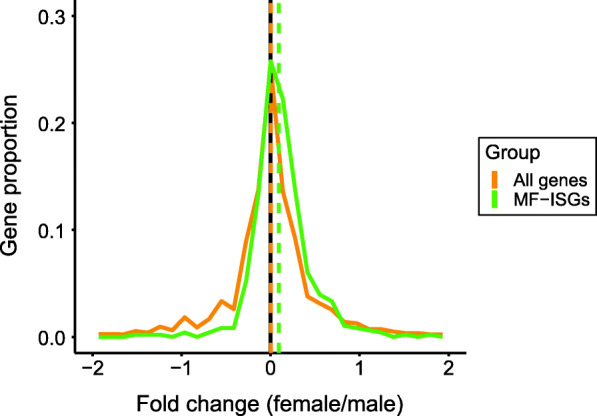
Female/male fold change (log2) distribution of genes related to IFN pathway from peripheral blood monocytes. Human orthologs of macrophage-specific IFN signature genes (MF-ISGs; 485 genes, green) and all other genes (20,602 genes, yellow). *p* = 0.00029 between the distributions (Welch’s two-sample *t* test)

Immune transcriptional sex dimorphism has been documented in unstimulated mouse macrophages, showing higher IFN responsiveness in females [19]. Based on this evidence and our pathway analysis results, we sought to further examine how the IFN pathways differ between males and females in human monocyte transcriptome. To this end, we first compared the monocyte sex-biased genes with *p* < 0.03 to a set of genes that were recently demonstrated to be upregulated by IFN in mouse macrophages (i.e., MF-ISGs, 601 genes). Of the 498 out of 601 MF-ISGs that have human orthologs, 485 genes were expressed in our monocyte samples. Out of the 360 female-biased genes in monocytes, 48 were also MF-ISGs (13.3%). In contrast, only six of the 205 (2.9%) male-biased genes were MF-ISGs (Fig. 2, Supplementary Table S2). Next, we assessed the expression of the entire set of MF-ISG human orthologs in our monocytes. The log_2_ fold change distribution of MF-ISGs was skewed toward females (mean = 0.09) and was significantly different from the symmetrical distribution of all other genes (20,602 genes, mean = 0.003) (two-sided *t* test *p* = 2.9 × 10^−4^; Fig. 3). Consistent with the pathway analysis, these results suggest that upregulation of IFN-response genes is more frequently observed in female than male monocytes under unstimulated conditions.

**Fig. 4:**
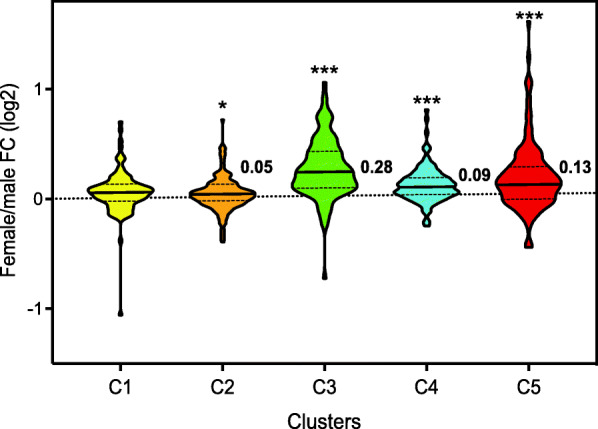
Violin plot of female/male fold change (log2) distribution of the genes in C1–C5 clusters of the IFN regulatory network constructed by Mostafavi et al. from peripheral blood monocytes. Each cluster denotes distinct function: C1-2 [RNA processing], C3 [antiviral effectors], C4 [metabolic regulation], and C5 [inflammation mediators or regulators]. The central line of each cluster indicates the median, and the bottom and top lines indicate the 25th and 75th percentiles, respectively. The clusters having mean fold changes significantly different from zero (one sample two-sided *t* test with Bonferroni correction) are marked with graded asterisks. * Adjusted *p* < 0.05; *** Adjusted *p* < 0.001

Despite conserved transcriptomic signatures of immune cell lineages between human and mouse, specific differences exist due to divergent evolutionary paths [33]. Moreover, monocytes have limited contribution to tissue-resident macrophages [34]. Thus, we applied a larger set of genes (623 genes), instead of MF-ISGs, that had been validated in various immune cells both from mouse and human and that cover more complicated IFN responses [29]. These genes are from an IFN transcriptional network consisting of 92 predicted regulators and 628 ISGs that was built based on co-expression in human and mouse responses [29]. Similar to the comparison with MF-ISGs, we assessed the expression of target ISGs in our monocytes. We identified expression of 623 genes out of the 628 ISGs in unstimulated monocytes and their log_2_ fold change distribution was significantly skewed to the female side (mean = 0.11), compared to that of all other genes (20,464 genes, mean = 0.002) (two-sided *t* test *p* = 2.2 × 10^−16^; Supplementary Fig. S1). We further examined sexual bias in expression of those ISGs based on the five clusters (C1–C5) that were parsed within the network and characterized by distinct functionalities; C1 and C2 enriched for RNA processing, C3 for antiviral effectors, C4 for metabolic regulation, and C5 for inflammation mediators or regulators [29]. While most of the clusters, except for C1, displayed significantly higher expression in females, the antiviral cluster C3 showed the strongest positive log_2_ fold change (median = 0.28, one sample two-sided *t* test Bonferroni-adjusted *p* < 2.2 × 10^−26^; Fig. 4), followed by the C5 cluster of inflammation (median = 0.13, Bonferroni-adjusted *p* = 7.6 × 10^−6^; Fig. 4). These results further confirm the higher baseline expression of IFN-responsive genes observed in female monocytes, particularly for the genes involved in antiviral and inflammatory responses, which may contribute to the phenotypic sex differences in autoimmunity.

## Discussion

Sex dimorphism in immune system is evidenced by differences in the prevalence and intensity of infectious diseases between the sexes [3] and the strong female-biased incidence of autoimmune diseases [4]. Compared to the marked phenotypic differences, sex differences at DNA sequence level are limited to the sex chromosomes and not apparent in autosomes [35]. This observation suggests an important role of higher-level molecular regulations such as transcriptional and epigenetic processes. In humans, a difference in transcriptome between males and females has been reported in the liver [17] and skeletal muscle [18] but also in whole blood [21, 23] or PBMC [20]. However, as peripheral blood contains several immune cell types, it is not clear whether the sex difference in the peripheral blood transcriptome results from differential immune cell type frequencies or different patterns of transcriptome in specific cell populations present between the sexes. Here, we compared transcriptional profiles of peripheral blood monocytes between male and postmenopausal female participants with chronic low-grade inflammation. Consistent with the observations in mouse macrophages [19], sex-biased differential gene expression was detected in our monocytes. Of note, the differentially expressed genes identified were greatly enriched for X and Y chromosome-linked genes. It has been reported that transcriptional sex differences in whole blood or PBMC of younger individuals, aged 18–40 years, was mainly attributed to autosomal genes [22]. Considering the significant reduction in estrogen levels after menopause and a gradually waning of androgen levels with aging in males, our findings may better represent the intrinsic genetic sex dimorphism.

Among the 22 female-biased differentially expressed genes, the majority were involved in essential features of innate immunity, immune cell activation and antigen-processing/presentation. These included *FCGR2B*, a member of Fc gamma (Fcγ) receptors. Those receptors are expressed on the surface of antigen-presenting cells (APC) such as monocytes, and mediate immune cell activation, phagocytosis, and production of inflammatory cytokines upon binding to immunoglobulin G [36]. Their functions are determined by the balance between activating and inhibitory Fcγ receptors [36]. Of note, not only the inhibitory *FCGR2B*, but also activating Fcγ receptors such as *FCGR1A* and *FCGR1C* tended to be expressed at higher levels in female monocytes, suggesting overall higher activation of monocytes in females than males with chronic inflammation. This is consistent with the immune sexual dimorphism observed in murine macrophages [19]. The higher expression levels of *UBA1* and *CD1E*, which processes antigens for the ubiquitin-proteasome system [37, 38] and presents lipid molecules as a major histocompatibility complex (MHC)-like protein [39], respectively, suggest a greater ability of female monocytes to present antigens, once infected by viruses, actively linking innate and adaptive immune systems. Higher antigen-presenting capability of APC has been reported in mouse splenocytes, possibly due to differential control of sex hormones [40].

The pathway analysis using IPA revealed that several immune-related pathways were upregulated in females in the unstimulated state, including IFN signaling as the top pathway for the sex differences. This may be indicative of a more vigilant innate immune defense in females under unstimulated condition. Interestingly, though the pathways identified by IPA need to be carefully interpreted in the context of cell type, most of the female-biased pathways include components involved in IFN signaling, suggesting an important role of IFN signaling in immune sexual dimorphism. For example, the female-biased Th1 pathway was characterized by upregulation of the genes related to MHC-II and IFN-STAT1 signaling, implying a propensity of female monocytes to foster Th1 differentiation [41]. This sex-based difference may account for the more robust Th1 immune responses observed in females than males in line with suppressive effects of androgens on key nodes of Th1 differentiation pathway including IL-12 and IFNγ production [42]. In contrast, the genes in the PD-1, PD-L1 pathway, which halts the development of T cells to minimize inappropriate autoimmune inflammation as an immune checkpoint [43], tended to be expressed higher in male than female monocytes (z-score = − 0.82). Taken together, monocytes from males and females may be in a differential basal state, especially during chronic inflammation, with regard to the balance between immune defense and its negative feedback system.

IFN, which plays a central role in initiating immune responses especially with antiviral effects [44], is also a key player implicated in the pathogenesis of a variety of autoimmune disorders such as SLE [26], the most female-biased disease with a 9:1 ratio of females to males [45]. In contrast to early SLE studies centered on the adaptive immune system, the paradigm has shifted with recent advances in the field of innate immunity, suggesting a crucial role of monocyte/macrophage abnormalities in the development of autoimmune responses [46]. To better understand how immune sex dimorphism in monocytes potentially contributes to the sex-biased susceptibility to autoimmunity, we further looked into IFN signaling. Using the gene set responding to IFN stimulation in mouse macrophages (MF-ISGs), which was recently published as part of the ImmGen project [29], we assessed sex-biased expression of human homologous genes in our monocytes. The overall expression of MF-ISGs was higher in females than males, as shown by the appreciable overlap with the female-biased genes and skewed fold changes toward females. In terms of functionality of ISGs [29], the most significant difference between the sexes was in genes of antiviral effectors, followed by genes of inflammatory mediators or regulators. These observations are largely consistent with the prior work that assessed the sexually differential expression of the same MF-ISG profiles in murine macrophages, where the ISGs for antiviral responses showed significant upregulation in female macrophages in both baseline and IFN-induced states while expression of the inflammation-regulating ISGs became significantly higher in females only upon IFN stimulation [19]. Our results suggest a more-primed basal-state innate immunity in females than males, especially in antiviral responses. In addition, our results of female-biased upregulation of Th1 pathway and male-biased PD-1, PD-L1 pathway suggest a greater transcriptional alertness of female monocytes to foster adaptive immune response. This may be a signature characteristic of human peripheral blood monocytes that is partially shared by murine macrophages from peritoneal cavity but not by those from the spleen, and microglia from the central nervous system [19].

The sex dimorphism in immune system, displaying a stronger IFN response of female monocyte/macrophages, appears to be conserved across a variety of species, including birds [19, 47]. Confirmation of human-mouse conservation of transcriptional sex dimorphism has previously been obtained through the significant overlap between the sex-biased genes of human CD14^+^ monocytes and murine macrophages [19]. Our results further confirm that human monocytes also exhibit the conserved sex dimorphic expression of IFN-responsive genes.

We observed an upregulation of the IFN signaling pathway in female monocytes using two different approaches. Due to the recruitment criteria confining study participants to men and postmenopausal women aged 50–75, it is unlikely that the immune sex dimorphism observed is confounded by other characteristics such as age [21] and menopausal status [20]. The frequencies of monocytes and other immune cell types were similar between males and females; therefore, our results may better reflect in vivo sex dimorphism of monocyte transcriptome. We observed higher expression of *TIFAB* and *CEACAM1* in females than males. *TIFAB* is an inhibitor of TNF receptor-associated factor (TRAF) mediated signal transduction down to NF-κB [48], and *CEACAM1* is a negative regulator of IL-6 signaling in response to LPS [49]. Reported in the parent study of our clinical trial [27], we found no differential expression between male and female monocytes of *TNFA*, *IL6*, and *MCP1*; the genes coding for cytokines and chemokines that are regulated by the TNF receptor pathways in both basal and LPS-stimulated conditions (Supplementary Table S3). These data suggest that other immune-related pathways may not have the transcriptional sex dimorphism as demonstrated in IFN signaling.

A limitation of our study was the relatively small sample size. In addition, we have not measured monocyte INFγ expression and secretion under LPS-stimulated conditions and therefore we do not know if the INFγ sex dimorphism is maintained under acute inflammatory stimuli. Further studies are needed to better characterize sex-based differences in monocyte-associated immune pathways.

In summary, sexual transcriptional differences in the immune system are present in basal-state human monocytes and are primarily associated with IFN-related signaling pathways.

### Perspective and significance

Sex differences in the development and progression of various immune-related diseases have been well documented but the molecular mechanisms are not well understood. Our data from human subjects support the previous findings from animals that indicated macrophage-specific IFN signaling as an important molecular effector of sex dimorphism in immune pathology. The present study suggests the importance of targeting IFN signaling in the treatment of sex-biased morbidity.

## Supplementary Information

**Additional file 1: Supplementary Table S1.** Sexually-based differentially expressed genes (SDEGs) in peripheral blood monocytes (n = 41, FDR < 0.1).

**Additional file 2: Supplementary Table S2.** Monocyte sex-biased genes (n = 565, p value < 0.03) and the overlap with IFN signature genes that were previously identified in mouse macrophages (MF-ISGs).

**Additional file 3: Supplementary Table S3.** mRNA expression and secretion of inflammatory cytokine/chemokines in peripheral blood monocytes with and without LPS stimulation.

**Additional file 4: Supplementary Figure S1.** Female/male fold change (log2) distribution of target genes of the IFN regulatory network, built by Mostafavi et al., from peripheral blood monocytes. The ISG target genes (623 genes, green) and all other genes (20,464 genes, yellow). p = 2.2 x 10-16 between the distributions (Welch’s two sample *t* test).

## Data Availability

Please contact the author for data requests.

## Abbreviations

APC: Antigen-presenting cells
BMDM: Bone marrow-derived macrophages
DHA: Docosahexaenoic acid
EPA: Eicosapentaenoic acid
FDR: False discovery rate
hs-CRP: High-sensitivity C-reactive protein
IFN: Interferon
ImmGen: The Immunological Genome Project
IPA: Ingenuity pathway analysis
IRF: Interferon regulatory factors
ISG: Interferon signature genes
MF-ISGs: Macrophage-specific interferon signature genes
MHC: Major histocompatibility complex
NF-κB: Nuclear factor kappa B
PBMC: Peripheral blood mononuclear cells
PCA: Principal component analysis
PD-1/PD-L1: Programmed cell death protein 1/programmed cell death-ligand1
SLE: Systemic lupus erythematosus
Th1: T helper type 1
TRAF: TNF receptor-associated factor
